# Design and optimization of highly sensitive multi-band terahertz metamaterial biosensor for coronaviruses detection

**DOI:** 10.1007/s11082-023-04906-6

**Published:** 2023-05-14

**Authors:** Zienab EL-Wasif, Tawfik Ismail, Omnia Hamdy

**Affiliations:** 1 National Institute of Laser Enhanced Sciences, Cairo University, Giza, 12613 Egypt; 2 Wireless Intelligent Networks Center (WINC), Nile University, Giza, Egypt

**Keywords:** **C**oronaviruses, THz sensing, Metamaterial biosensor, Flu detection

## Abstract

This study presents the design and characterization of a highly Q-Factor and ultrasensitive THz refractive-index-based metamaterial biosensor for detecting coronaviruses at electronic infusion device (EID) concentrations $0.01$ and $1000$. The proposed biosensor is constructed using a gold plane perforated by a star shape. Moreover, the developed structure is polarization insensitive due to the rotatory symmetry and is angularly stable up to 90°. The proposed biosensor achieves near-perfect absorption at $1.9656$ THz and $3.3692$ THz. The full width at half-maximum is $5.276\%$ and $0.641\%$ comparative to the absorption frequency. In addition, the estimated free space absorptivity is 97.2% and 99.1% with a Q-Factor of 19.08 and 155.98 at 1.9656 THz and 3.3692 THz, respectively, when transverse electromagnetic mode (TEM) was selected. The perforated star-shaped was evaluated for IBV (Family of COVID-19) regarding frequency deviation, sensitivity, and figure of merit. Results show that at 1.9656 THz, the proposed design gives 30.8 GHz, 940.49 GHz/RIU, and 8.6, respectively, for 0.01 (EID/5 µL concentration) and 4.4 GHz, 2200 × 10^3^ GHz/RIU, and 20,215.014, respectively at 1.9612 THz for 1000 (EID/5 µL concentration). Although the obtained results demonstrate the efficiency of the proposed THz metamaterial biosensor in coronavirus detection, it has also been extended for other types of viruses, including H5N1, H5N2, H9N2, H4N6, and FAdV, based on the slight variations in their refractive indices. Additionally, the influence of the design parameters is optimized in order to achieve better performance.

## Introduction

Significant studies for developing credible and timely differentiation of infectious viruses using standard methods began at the end of the twentieth century, when intermittent diffuse epidemics from emerging viruses, such as HIV, severe acute respiratory syndrome (SARS), and the Middle East respiratory syndrome coronaviruses (MERS), pandemic influenza H1N1, Ebola, and Zika, now SARS-CoV-2 virus-related respiratory syndrome, i.e., COVID-19 (Ahmadivand et al. 2021; Calvo-Lozano et al. 2022; Di Fabrizio et al. 2021; Kuppuswamy et al. 2020). SARS-CoV-2 was declared a pandemic after rapidly spreading worldwide, increasing the number of critical cases and death rates, particularly among chronic disease patients (Huang et al. 2020). The standard method for virus detection is the Reverse Transcriptase -Polymerase Chain Reaction (RT-PCR) test, which is considered an invasive method in addition to other disadvantages such as complexity, delay, and expense (Khaja et al. 2021). Therefore, owing to the recent advances in optical biosensing technologies, alternative methods for rapid, accurate, and reliable detection are proposed. Optical biosensing technologies offer a promising alternative to RT-PCR due to their non-invasive nature, simplicity, and potential for real-time detection. These technologies utilize various sensing mechanisms, such as surface plasmon resonance, fluorescence, and photonic crystals, to detect susceptible and specific viral particles.

Photonic crystal fiber (PCF) is a form of refractive-index-based optical biosensors in which light is is confined within core of the PCF structure (Mitu et al. 2022). PCF’s have shown promising performance in sensing different biological materials including different blood components (Ahmed et al. 2019a) and blood plasma (Ahmed et al. 2019c) due to its applicabiltiy in tailored and flexible microstructures. They have also been utilized in gas (Paul et al. 2018) and alchol (Ahmed et al. 2019b) sensing in addition to diesel adulteration detection (Jabin et al. 2022). Over the past decade, PCF based on split ring resonance (SRR) has developed as one of the most reliable, robust, and effective sensing techniques (Ahmed et al. 2019c). It was effectively utilized in detecting biomolecules and organic chemicals (Yang et al. 2021) in addition to hemoglobin sensing (Jabin et al. 2019). SPR is occurred when the incident electro-magnetic waves oscillate free electron on the metal-dielectric interfaces (Chow et al. 2016).

It is worthy to mention here that, sensor delay is important factor affecting stability of the optical fiber sensors (Li et al. 2021b). However, one great advantage of THz metamaterials refractive-index-based biosensors that they have rapid sensing characteristics especially in virus detection (Akter et al. 2021). Several THz spectral studies over the past few decades have shown that many biomaterials, such as proteins and viruses, appear to either transmit or absorb THz waves (Hou et al. 2021; Veeraselvam et al. 2021a). Terahertz time-domain spectroscopy (THz-TDS) has been the subject of extensive studies for its potential for detecting microorganisms (Park et al. 2017). This has led to the development of THz spectroscopy as a promising tool for biomedical research, including disease diagnosis and drug discovery. With its non-invasive and label-free nature, THz spectroscopy has the potential to revolutionize the way we study biological systems. Recent years have seen growth in the application of THz radiation-based biomedical sensors for diagnostic purposes (Ahmed et al. 2019a). The amount of THz radiation that the virus sample absorbs varies with its refractive index. It gives information about the components that are present in the sample of corona as well as the category of other flu viruses or any other biological sample. This method could be used to quickly and painlessly tell the difference between different types of viruses, which could help scientists make better treatments. However, further research is needed to fully understand the potential applications of THz radiation in virus detection.

Terahertz (THz) radiation, ranging from $0.1$ to $10$ THz, has attracted the attention of researchers due to its non-invasive and non-ionizing radiation characteristics. It has several applications in the fields of biomedical spectroscopy and imaging., as well as finding cancer cells in their early stages and diagnosing skin diseases because it can get deep into tissues without hurting them. The THz spectrum is different because it can reach deeper into surfaces using only a small amount of energy. This makes it a promising tool for non-invasive medical imaging and could revolutionize how we diagnose and treat diseases in the future. Due to the lower toxicity of THz radiation, it can be safely used for diagnostic purposes. These radiations have been the subject of much research and investigation in a variety of fields, including spectroscopy (de Almeida et al. 2021; Jepsen et al. 2011), imaging (MacPherson et al. 2013; Sanphuang et al. 2015; Watts et al. 2014), and sensing (Amin et al. 2021; Li et al. 2021a; Vafapour et al. 2021). Recently, metamaterials have been extensively used to structure miniaturized THz compositions and equipment. Artificially structured materials with frequent mineral or semi-mineral unit cells on the sub-wavelength scale, such as metamaterial biosensing chips, have garnered significant interest in recent decades because of their peculiar electromagnetic properties. These properties include the ability to act as a perfect absorber, invisibility cloaking, negative refractive index, and superlens (Areed et al. 2018; Monti et al. 2015; Schurig et al. 2006; Xu et al. 2013).

The metamaterials can progress the precision of field-matter interaction characteristics within robustly bounded resonance fields and severe spectral characteristics, strengthening biosensing sensitivity for biological materials. Therfore numerous studies have been reported in devloping THz metamaterials sensors to be utlizied in spectroscopic sensing of various bilogical materials such as proteins (Hou et al. 2021; Wang et al. 2021) and viruses (Cheng et al. 2018) in addition to cancer biomarkers (Geng et al. 2017). In the research literature, THz metamaterial absorbers are frequently used for material characterization. These absorbers are particularly useful for non-destructive testing, as they can detect small variations in the tissue's properties. Additionally, they have potential applications in medical imaging and DNA screening. Park et al. (2017) described THz metamaterials as an efficient sensing platform for observing low-density viruses like PRD1 and MS2. Both viruses are typical of their subtypes because they affect single-stranded RNA and double-stranded DNA. Lee et al. (2017) studied nano-metamaterial sensor chips integrated with THz spectroscopy to learn more about the THz optical features of various AI viruses. Their findings could potentially lead to the developing of new diagnostic tools for detecting AI viruses.

Other researchers investigated a nanoscale metamaterial reflector made of fano-resonances consisting of a graphene H-shaped antenna embedded in the middle of an InSb semiconductor layer (Keshavarz and Vafapour 2019). They numerically demonstrated that the proposed nano biosensor worked as an effective sensing platform for detecting Avian Influenza (AI) viruses such as H1N1, H5N2, and Hviruses9N2, which have different complex values of the refractive indices (RI). It achieved the maximum sensitivity is 540 GHz/RIU. Cheng et al. (2018) achieved Jerusalem cross apertures’ metamaterial absorber based on the strongly confined spoof surface plasmon polaritons resonance mode in THz regime for different subtypes and protein concentration biosensing of AI viruses. State-of-the-art research involves the development of THz metamaterials with split-ring resonators characteristics (Geng et al. 2022; Islam et al. 2022; Nourinovin and Alomainy 2021). The authors proposed a metamaterialstructure that exhibits electromagnetically induced transparency-like (EIT-like) Fano resonance. The structure is consisting of asymmetric split-ring resonators in which increasing the asymmetry results in transparency window with a peak at $1.94$ THz.

In the present paper, an ultrasensitive THz biosensor is developed and analyzed for coronaviruses detection. The proposed metamaterial unit cell is a sub-wavelength element that is used to construct a near-infinite periodic array. The sensor dimensions are studied in order to achieve better reliability and performance in terms of sensitivity (S), and Figure of Merit (FoM) with two EID concentrations of $0.01$ and $1000$. The average absorptivity is 97.2% and 99.1% at 1.9656 THz and 3.3692 THz, respectively. The proposed sensor has a footprint of 0.609 λ_eff_ × 0.609 λ_eff_ where λ_eff_ is the wavelength calculated at the operating frequency of 1.9656 THz. The full-width at half-maximum (FWHM) is 5.276% and 0.641% comparative to absorption frequency. The maximum theoretical sensitivity is 968 GHz/RIU and 264 GHz/RIU at 1.9656 THz and 3.3692 THz. The proposed THz biosensor is polarization insensitive due to the rotatory symmetry. It is angularly stable up to 90° and keeps its efficient performance regardless of the fabrication tolerance of ± 5%. The design and analysis for all parameters of the THz biosensor are performed using Computer Simulation Technology (CST) Studio Suite 2020 (Das and Varshney 2021; Veeraselvam et al. 2021b). The theoretical values of the refractive index of the corona (COVID-19) and category of other flu viruses ($\mathrm{H}5\mathrm{N}1,\mathrm{ H}5\mathrm{N}2,\mathrm{ H}9\mathrm{N}2,\mathrm{ H}4\mathrm{N}6,\mathrm{ FAdV}$) are used to evaluate the sensing performance of the proposed sensor. In addition, the polarization, angular stability and the sensitivity of the proposed biosensor are analyzed and compared with the traditional sensors of a single resonant peak, the sensor with two resonant frequencies can significantly reduce the measurement errors and achieve higher accuracy.

### Biosensor design

**Fig. 1:**
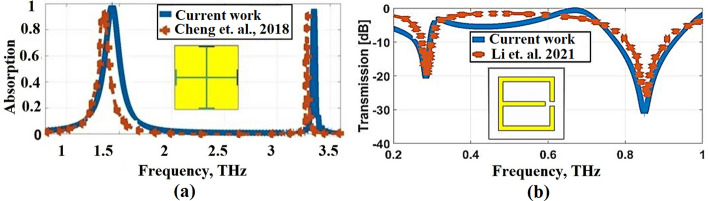
Results validation in comparison with, **a** Absorption characteristics of Cheng et al. (2018) and the presented results, **b** transmission results of Li et al. (2021a)

Several metamaterial structures, such as split rings, strips, rings, and the Jerusalem cross structure, can achieve perfect absorption. The Jerusalem cross structure is regarded as a classic absorber structure because of its excellent absorption capabilities (Cheng et al. 2018; Pan et al. 2021). In this paper, we propose an ultrasensitive and selective THz metamaterial absorber structure that simplifies the Jerusalem cross structure as illustrated in Fig. 1. The absorber structure proposed in this study is composed of a gold plane that has been perforated in star-shaped. It is a standard sandwich construction consisting of a metal pattern as the top layer, silicon dioxide as the intermediate dielectric layer, and a metal plane as the bottom layer, with the metal pattern serving as the top layer.

For evaluating the accuracy of the proposed simulation results, a comparison has been made with THz biosensing metamaterial absorber that has been proposed by Cheng et al. (2018), and a metamaterial THz biosensor that has been that has been fabricated and simulated by Li et al. (2021a). This comparison was performed to guarantee that the fabricated sensor will produce equivalent simulation results. Therfore, our simulation setup is supposed to successfully reproduce the same results guaranteeing the correctness of the theoretical analysis. The full-wave EM solver in CST Microwave Studio uses an accuracy setting of $1{0}^{4}$ and a mesh size of $15$ lines (cells) per wavelength in order to achieve the desired accuracy. Figure 1 presents the results proposed by Cheng et al. (2018) (Fig. 1a) and Li et al. (2021a) (Fig. 1b) compared with our simulation results. It can be noticed that a good agreement is achieved between our results and those reported in Cheng et al. (2018) and Li et al. (2021a) experimentally and theoretically which ensures the high accuracy of our calculations.

### Construction

The CST uses a finite integration technique (FIT) to simulate and develop the geometrical parameters of the proposed THz biosensor in order to get the highest possible absorption peak and sensitivity. The FIT method is a powerful tool for analyzing the electromagnetic behavior of complex structures. It allows for the simulation of various scenarios and enables researchers to optimize the design of THz biosensors for specific applications. Parameter sweep with a very tiny ring was employed in order to achieve an optimal configuration of the geometrical parameters s $a, b$ and $c$ to obtain maximum absorptivity with very sharp resonances (Veeraselvam et al. 2021a, 2021b).

**Fig. 2:**
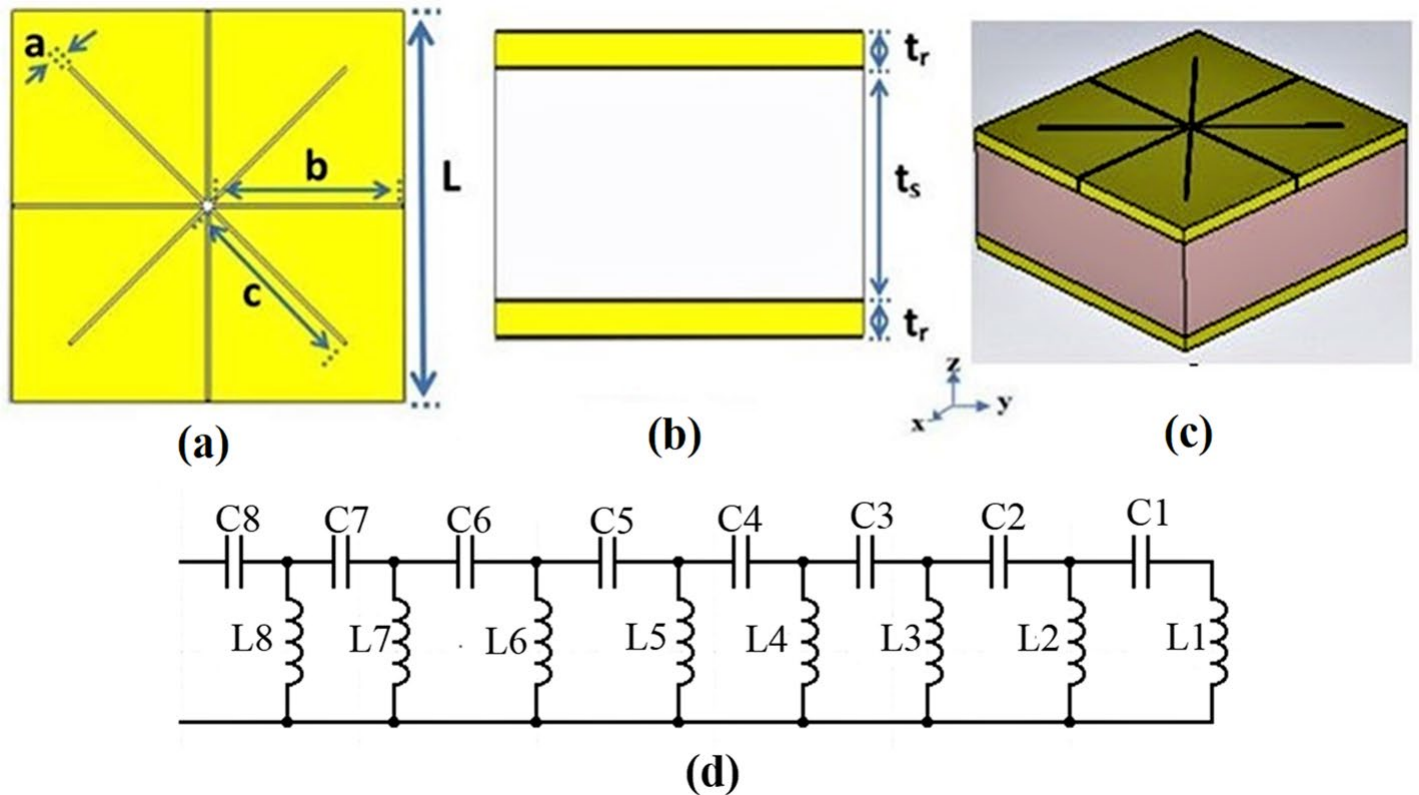
Proposed THz sensor, **a** Top view, **b** Side view, **c** Perspective view, **d** LC circuit of the proposed THz sensor

The proposed sensor with its top, side and perceptive views are illustrated in Fig. 2a–c, respectively. The optimized footprint of the proposed biomedical sensor is $64.15\left(\mathrm{L}\right)\mathrm{ \mu m}\times 64.15\left(\mathrm{L}\right)\mathrm{ \mu m}$ (Cheng et al. 2018). The unit cell comprises an upper gold plane with star-shaped holes and a gold packing plate separated by a conventional silicon dioxide layer. The geometrical parameters of the metamaterial absorber are: $\mathrm{a}=0.6\mathrm{ \mu m}$, $\mathrm{b}=31.7\mathrm{ \mu m}$, $\mathrm{c}=33.7\mathrm{ \mu m}$, slot depth and upper gold thickness ${\mathrm{t}}_{\mathrm{r}}=2\mathrm{ \mu m}$ is equal to gold packing plate thickness, and substrate thickness, ${\mathrm{t}}_{\mathrm{s}}=13\mathrm{ \mu m}$. Gold is utilized for the start shaped layer on the top surface. The gold layer is modeled as a lossy medium with a frequency independent conductivity = 4.09 × 10^7^ S/m (Ma et al. 2020; Tao et al. 2008).

The circuit model of the suggested design is presented in Fig. 2d. The model was created based on the equivalent circuit theory as the perforated star-shaped and the mineral base plate form a current loop (Ma et al. 2020). The gaps form equivalent capacitances are defined by C_1_ to C_8_, the metal strips form equivalent inductances are L_1_to L_8_. Effective capacitances of the structure come from the top analyte and the dielectric layer sandwiched between the two mental layers are represented by C_sensor_ and C_d_, respectively. The resistance in the RLC circuit is a representation of the energy that is being dissipated. In the proposed study, we are not calculating the attenuation of radiation. However, the resonance frequency is of particular relevance to us. In this situation, it is possible that the resistance would be neglected (Ullah et al. 2020). Within the context of the LC model, the length between gaps is 600 nm which could be presented by the capacitors. Moreover, the upper layer is extremely thin, and the gap between the edges in the central point is far larger than the distance between the edges themselves. Total inductance will be ${L}_{e}$, total device capacitance ${C}_{e}$ and a sensing capacitance C_sensor_. The value of C_sensor_ varies with the refractive index n_s_ and thickness H_s_ of the surface analyte. Furthermore, the resonance frequency of the sensor can be expressed by:

1 $$F\mathrm{r}=\frac{1}{2\pi \sqrt{{L}_{e}+{C}_{e}+{C}_{sensor}}}$$

The equivalent device capacitance C_e_ will be much smaller than C_sensor_ if the relative dielectric constant of dielectric is small and the gaps between the grooves at the resonance frequencies. So that C_sensor_ will take charge of equivalent capacitance of the sensor, and further brings a prominent shift of resonance frequency when there is a minor variation of analyte refractive index providing high sensitivity (Ma et al. 2020).

The transmission (T), reflection (R), and absorption (A) characteristics are analyzed using CST’s finite integration technique with periodic boundary conditions along $\mathrm{x}$ and $\mathrm{y}$ directions CST/FIT is used to look at the transmission $(T)$, reflection $(R)$, and absorption $(A)$ characteristics with periodic boundary conditions in the $x$ and $y$ directions for TE and TM modes, electric and magnetic boundaries in the x and y directions for TEM mode, and open boundaries (add space) in the $z$-direction. In order to excite the proposed sensor, a THz wave propagating in the z direction is used. The absorption properties of the proposed sensor are assessed by analyzing scattering parameters. The scattering parameters are also used to determine the reflection and transmission coefficients of the sensor, which are important in characterizing its performance and optimizing its design. The following relation is used to calculate an estimate of the absorptivity, or absorbed power:

2 $${\text{A}}\left(\upomega \right) = 1{-}{\text{R}}\left(\upomega \right){-}{\text{T}}\left(\upomega \right) = 1 - \left| {{\text{S}}_{{11}} } \right|^{2} - \left| {{\text{S}}_{{21}} } \right|^{2}$$

where S_11_ and S_21_ are the measured reflection coefficient and transmission coefficient respectively. $\mathrm{T}\left(\upomega \right)=0$ due to the gold layer beneath the substrate, which prevents the THz wave propagation along the rear side. Then, the calculation formula of the absorption (1) can be simplified to,

3 $$\mathrm{A}(\upomega )=1-\mathrm{R}(\upomega )=1-|{\mathrm{S}}_{11}{|}^{2}$$

when $\mathrm{R}(\upomega )=0$, the absorber achieves perfect absorption. The quality factor (Q) is a good measurement for evaluating the performance of a sensor. According to the formula $\mathrm{Q}={\mathrm{F}}_{\mathrm{r}}/\mathrm{FWHM}$ (Veeraselvam et al. 2021a), the value of Q is determined by the values of resonant frequency (${\mathrm{F}}_{\mathrm{r}}$) of the absorber and full width half maximum (FWHM) of the absorption peak. Another performance index of the narrowband absorber is the full width at half-maximum of the absorption peak comparative to the absorption frequency, which can be appointed as FWHM$/{\mathrm{F}}_{\mathrm{r}}\times 100\mathrm{\%}$. The narrowband absorber with a low comparative bandwidth of full width at half-maximum has serious application horizons in the fields of sensing and photoelectric detection (Pan et al. 2021).

### Evolution

**Fig. 3:**
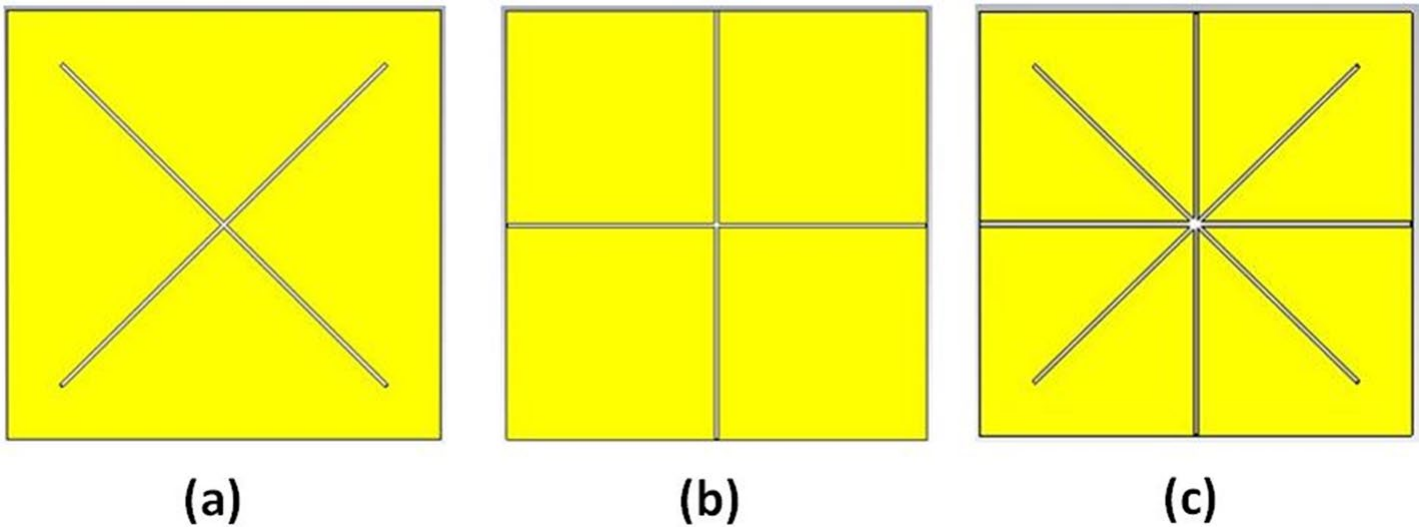
Evolution of the proposed THz sensor, **a** Resonator 1, **b** Resonator 2, **c** Integrated Resonator

**Fig. 4:**
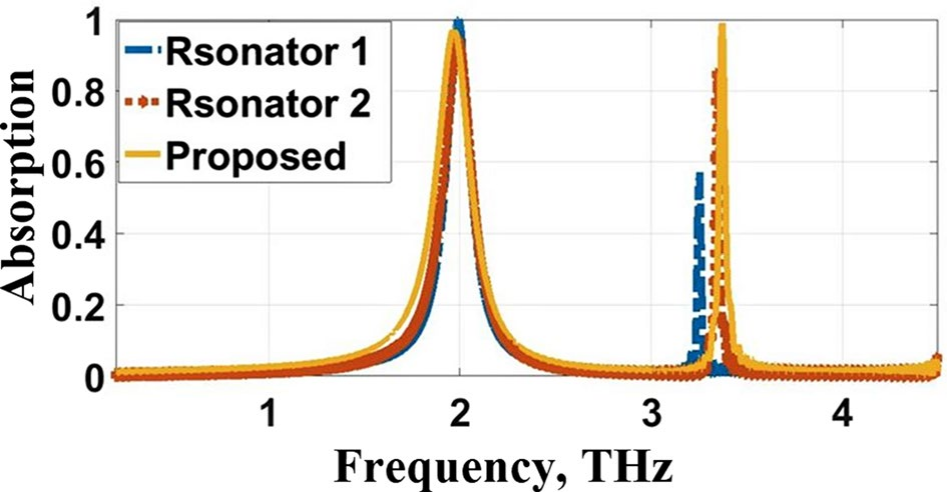
Absorption characteristics during different stages of evolution

**Fig. 5:**
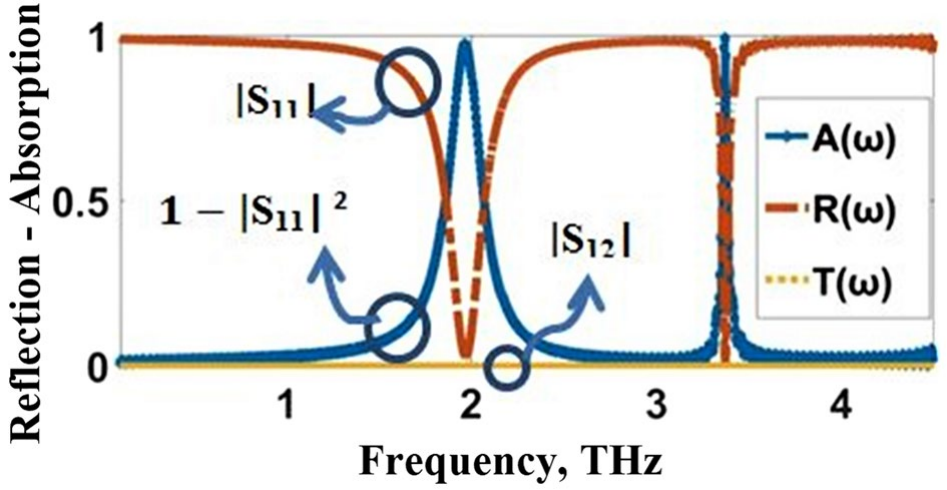
Reflection, Transmittance, and Absorption characteristics of the proposed THz sensor

The evolution of the proposed THz biosensor as well as the corresponding absorption characteristics, are illustrated in Figs. 3 and 4, respectively. The proposed THz sensor is developed from a gold plane perforated by a cross-shaped resonator synthesized, as shown in Fig. 3a with side length $\mathrm{c}=33.7\mathrm{ \mu m}$ and width $\mathrm{a}=0.6\mathrm{ \mu m}$. It operates at $1.992$ THz, and $3.2504$ THz offers $97.9\mathrm{\%}$ and $57.04\mathrm{\%}$ absorptivity. The gold plane is perforated by a plus-shaped resonator as described in Fig. 3b with length $\mathrm{b}=31.7\mathrm{\mu m}$ and width $\mathrm{a}=0.6\mathrm{\mu m}$. It operates at $1.992$ THz at $3.3428$ THz offers $94\mathrm{\%}$ and $84.57\mathrm{\%}$ absorptivity. The resonant frequencies are $1.9656$ THz and $3.3692$ THz with an exceedingly high absorptivity of $97.29\mathrm{\%}$ and $99.06\mathrm{\%}$, as described in Figs. 3c and 4. The absorption spectra and the reflection and transmission characteristics are plotted in Fig. 5. From the figure, it can be inferred that the proposed sensor has zero transmission characteristics with incredibly low reflection property and extremely high absorptivity at $1.9656$ THz and $3.3692$ THz. The gold layer beneath the substrate prevents the THz wave propagation along the rear side (i.e. T (ω) = S_12_^2^ = 0).

**Fig. 6:**
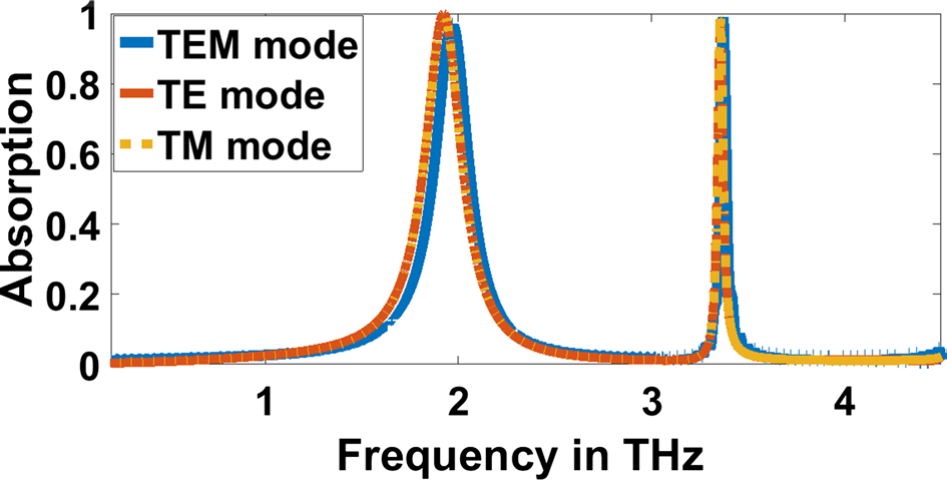
Polarization stability characteristics of the proposed sensor for all modes

**Fig. 7:**
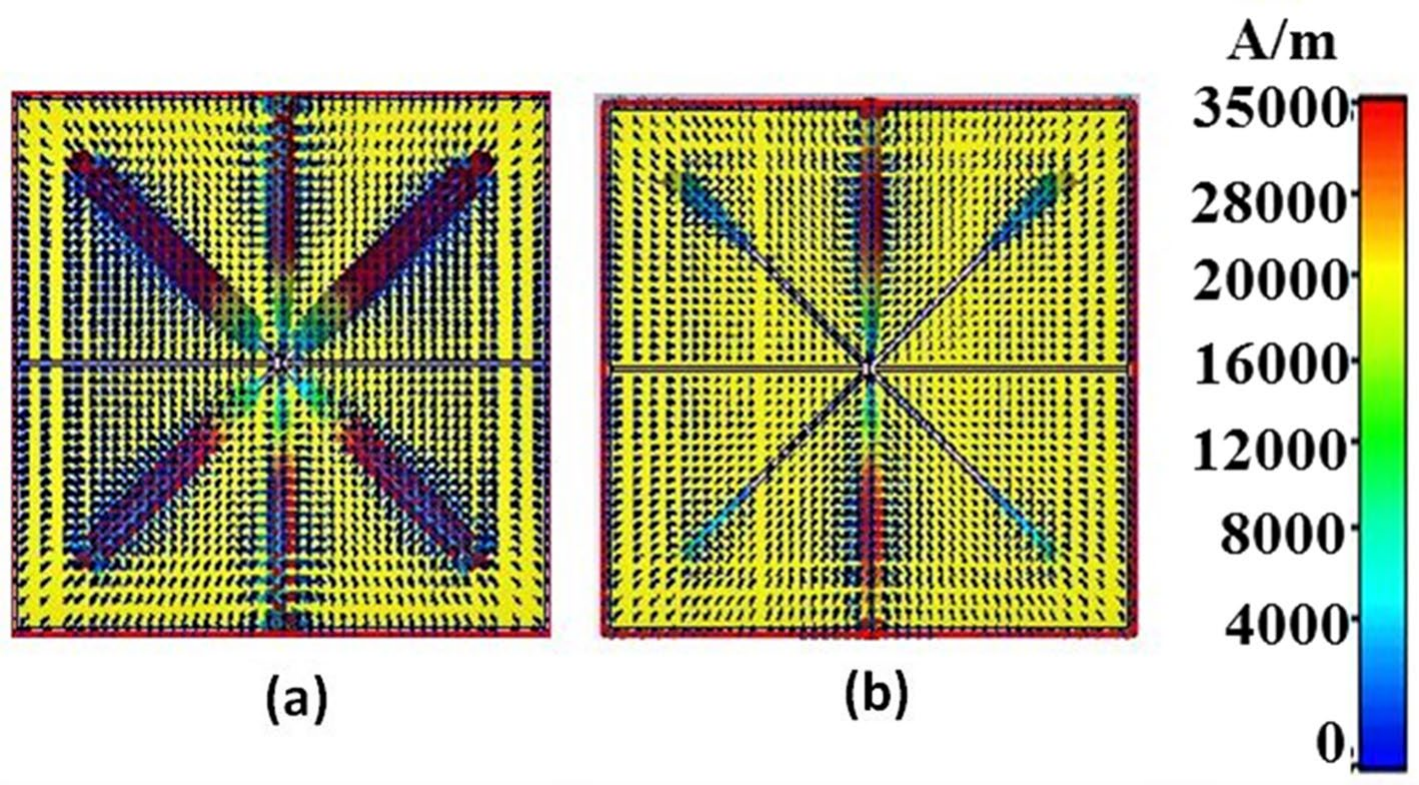
Surface plasmon density for TEM mode at, **a** 1.9656 THz, **b** 3.3692 THz

The sensor is subject to THz radiation along the $\mathrm{z}-$ direction. The polarization stability under normal incidence $\uptheta ={0}^{\mathrm{o}}$, is evaluated for transverse electric (TE) and transverse magnetic (TM) modes with $\upphi ={0}^{\mathrm{o}}$ and $\upphi =9{0}^{\mathrm{o}}$, and for TEM mode. The rotational geometry of the proposed structure provides closely matched absorption characteristics for TEM, TE, and TM mode of operation, as illustrated in Fig. 6 and Table 1. This signifies the polarization-insensitive behavior of the sensor for the incoming polarized THz waves. To further validate the polarization stability, the surface plasmon density corresponding to TEM mode at $1.9656$ THz and $3.3692$ THz of operation is described in Fig. 7a and b respectively. From the figure, it is inferred that all the electromagnetic power is concentrated in the grooves at the resonance frequencies, which provides an indirect process to discriminate the dielectric discrepancy in the virus detection. While the plasmon density is high at $1.9656$ THz, it is chosen as the target biosensing mode for the coronavirus.

**Table 1:** Absorption characteristics, resonance frequency and FWHM for all modes

Mode	Absorption	F_r_ (THz)	FWHM	Q-factor
TEM	0.972	1.9656	0.1037	19.08
	0.991	3.3692	0.0216	155.98
TE	0.999	1.9216	0.127	15.13
	0.967	3.356	0.0174	192.87
TM	0.999	1.9216	0.127	15.13
	0.983	3.3539	0.01779	188.527

**Fig. 8:**
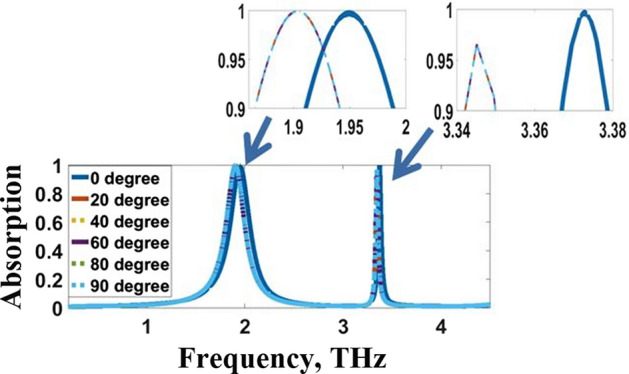
Angular stability characteristics of the proposed sensor

Furthermore, the leverage of the THz waves with perverse incidence on the absorption merits is elaborated and offered in Fig. 8. The angular constancy is weighted as the range of angles $\uptheta$ over which the sensor is estimated to exhibit identical countenances with frequency deviation less than $0.05$ THz and absorptivity greater than $95\mathrm{\%}$ for both resonance frequencies. As the angle $\uptheta$ increases, the absorption decreases, and the resonant frequency attain a blue shift. Thus, the proposed THz sensor is angularly independent for angles up to $\uptheta =9{0}^{\mathrm{o}}$. Since the proposed structure is polarization independent, the oblique incidence resulted in identical responses for TEM, TE, and TM modes of operation. Consequently, the proposed sensor is insensitive to polarization and angularly independent (*i.e.* it is not affected by the source position). This feature makes the proposed structure appropriate for rapid detection. These values of the proposed sensor are better than the previous results in Veeraselvam et al. (2021a). In Veeraselvam et al. (2021a), the THz sensor is angularly independent for angles up to $\uptheta =6{0}^{\mathrm{o}}$ with frequency deviation less than $0.1$ THz and absorptivity greater than $80\mathrm{\%}$.

## Results and discussion

### Sensitivity estimation

**Fig. 9:**
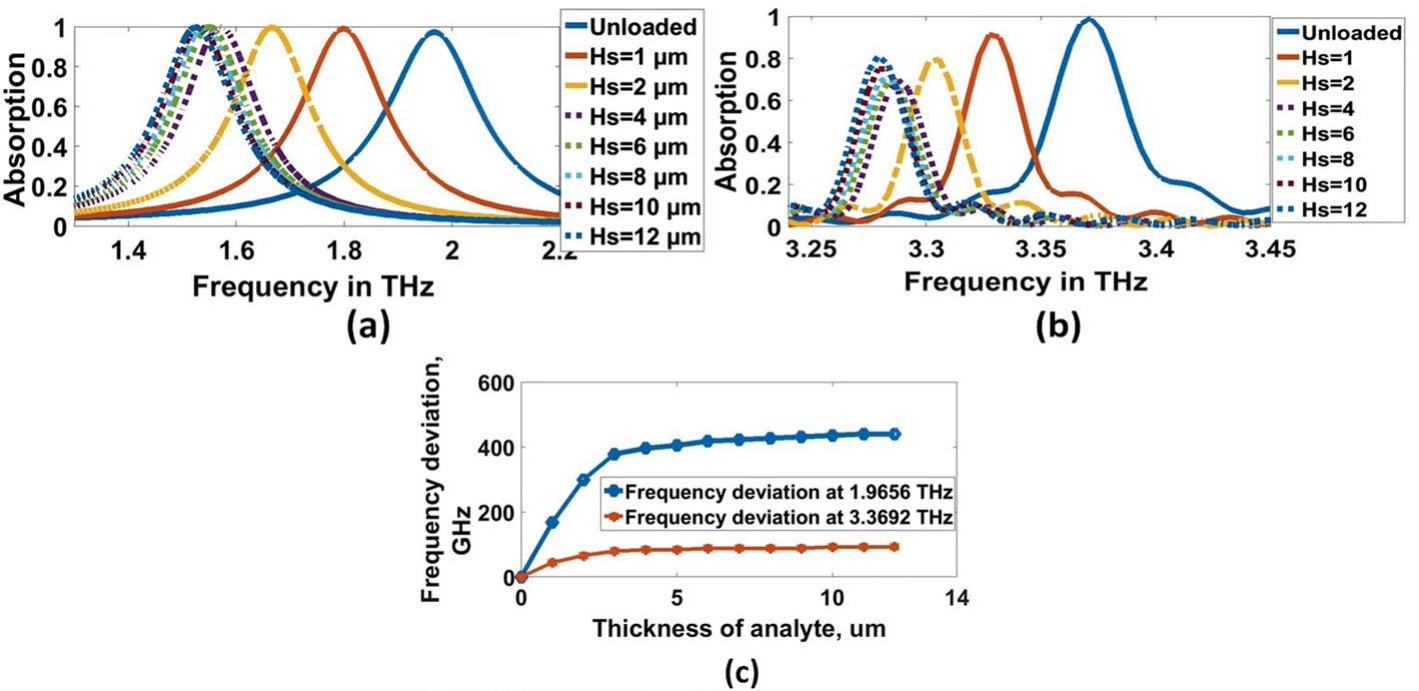
Absorption charactertics for various the thickness of analyte at, **a** 1.9656 THz and, **b** 3.3692 THz, respectively, **c** Frequency deviation of changing the thickness of analyte

**Fig. 10:**
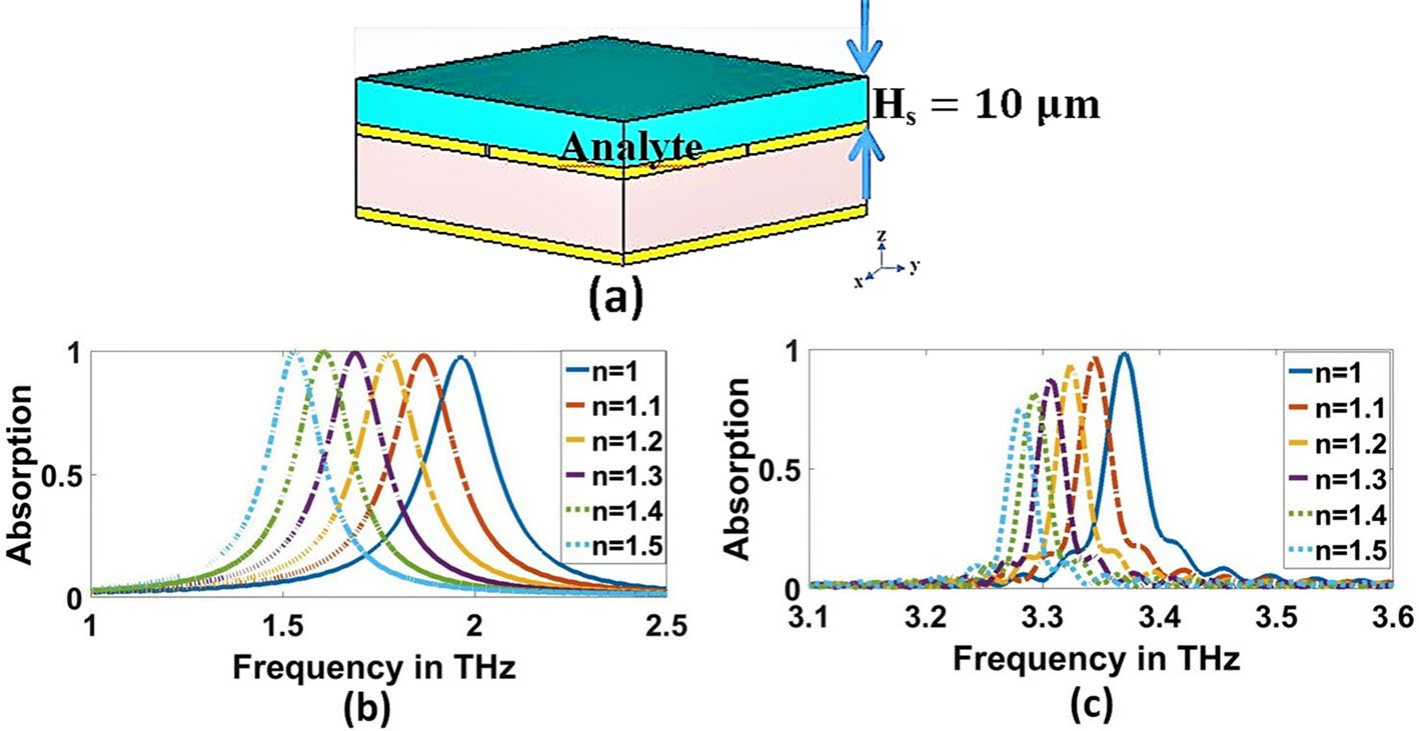
**a** Experimental setup for sensitivity estimation, **b** and **c** Sensor performance for various materials characterized by the different indexes of refraction at1.9656 THz and 3.3692 THz, respectively

The refractive index (RI) sensing applications of the proposed THz biosensor structure are among the most impressive applications of the proposed THz biosensor structure. The proposed THz biosensor can determine the refractive index of the medium in which it is placed. The performance of the THz biosensor for various sample thicknesses is evaluated and illustrated in Fig. 9a and b at $1.9656$ THz and $3.3692$ THz, respectively with a refractive index of $1.5$. The red shift of $167.2$ GHz and $44$ GHz is observed when the biochemical analyte of thickness $1\mathrm{ \mu m}$ is loaded into the proposed THz biosensor at $1.9656$ THz and $3.3692$ THz, respectively. The estimated average frequencies of deviation are $358.4308$ GHz$/\mathrm{\mu m}$ and $75.8154$ GHz$/\mathrm{\mu m}$ at $1.9656$ THz and $3.3692$ THz, respectively. when the thickness of the analyte is varied from $1$ to $12\mathrm{\mu m}$ as shown in Fig. 9c. Furthermore, the absorption spectrum is shifted by increasing the refractive index of the analyte. The refractive index biosensor that has been proposed is highly sensitive to fluctuations in the refractive index of the surrounding environment. As illustrated in Fig. 10, the absorption spectra of the THz biosensor are exhibited with varied refractive indices of the surrounding medium.

### Effect of materials

The sensitivity (S) can then be adjusted by changing the refractive index of the surface analyte once the structural characteristics and materials of the metamaterial have been specified, which is appointed as $\mathrm{S}=(\mathrm{\Delta F})/(\mathrm{\Delta n})$ GHz/RIU (Keshavarz and Vafapour 2019), where $\mathrm{\Delta n}$ is the difference in the reflective index between free-space and the analyte. In contrast, $\mathrm{\Delta F}$ is the peak frequency shift. For the non-destructive analyte, the refractive indices were selected by $\mathrm{1.0,1.1,1.2,1}.\mathrm{3,1.4,1.5},$ and $1.6$, covering the metamaterial surface. Furthermore, the analyte thickness was selected by $10\mathrm{\mu m}$, as shown in Fig. 10a.

**Fig. 11:**
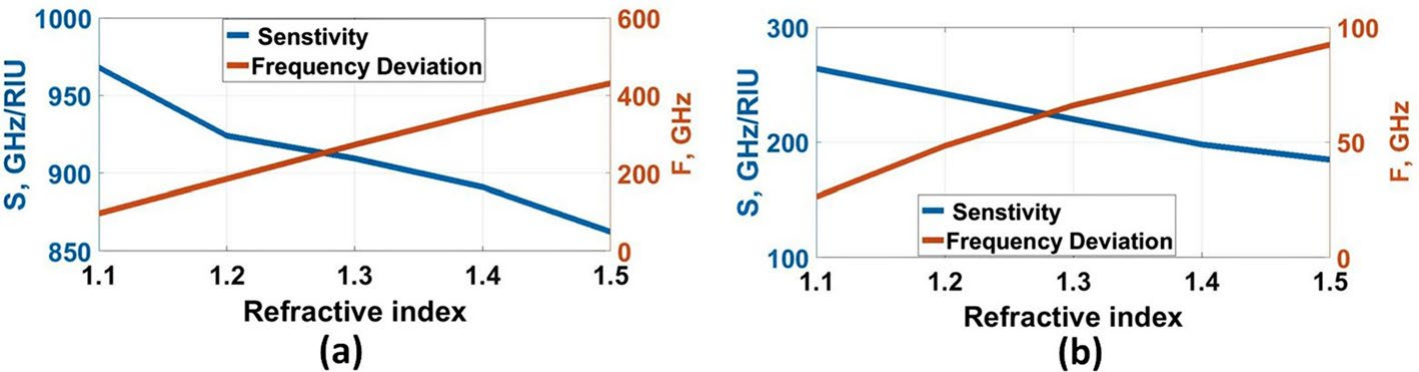
Sensitivity and frequency deviation for various indexes of refraction at, **a** 1.9656 THz and, **b** 3.3692 THz, respectively

The absorption characteristics for each resonance frequencies are shown in Fig. 10b and c. We can observe that, at $1.9656$ THz, there is no degradation in absorption, but there is degradation in absorption at $3.3692$ THz due to large spoof surface plasmon polaritons (SSPPs). So, lower resonant can be chosen as the target biosensing SSPPs for virus detection. Additionally, the resonance peak shifts to a lower frequency for both resonance frequencies with the refractive index increase. This shift can be physically explained as follows; the increase in refractive index increases the effective capacitance (*C*_*e*_) leading to a decrease in the quality factor (Q) of the sensor resulting in resonant frequency reduction (Veeraselvam et al. 2021a). Changing the refractive index from $1.0$ to $1.1$ ($\Delta n=0.1$) leads to a frequency shift by $96.8$ GHz. Therefore, the corresponding sensitivity $(S$) becomes $968$ GHz RIU. The results of the sensitivity for the proposed refractive indices are presented in Table 2. Figure 11a and b shows the sensitivity and frequency deviation at $1.9656$ THz and $3.3692$ THz, respectively. for lower resonant, the average sensitivity and frequency deviation are 898.511 (GHz/RIU) and 307.3 GHz, respectively. These values in the higher resonant are 212.9112 (GHz/RIU) and 68.9333 GHz, respectively. The sensitivity and frequency deviation are a constant linear variation for both resonant.Thus, the frequency deviation study is more interesting for virus detection.

**Table 2:** Sensitivity and frequency shift of various refractive indexes

RI	F_r_ (THz)	Δf (GHz)	S (GHz/RIU)
1	1.9612	–	–
	3.3692		
1.1	1.8644	96.8	968
	3.3428	26.4	264
1.2	1.7764	184.8	924
	3.3208	48.4	242
1.3	1.6884	272.8	909.333
	3.3032	66	220
1.4	1.6048	356.4	891
	3.29	79.2	198
1.5	1.53	431.2	862.4
	3.2768	92.4	184.8
1.6	1.4594	501.8	836.333
	3.268	101.2	168.667

**Fig. 12:**
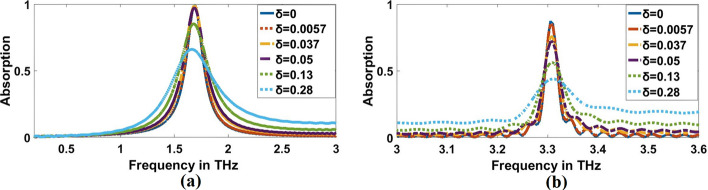
Effect of dispersion on the proposed sensor for refractive index 1.3 at, **a** 1.9656 THz and, **b** 3.3692 THz, respectively

Figure 12 illustrates the impact of dispersion on the absorption properties of a sample with a refractive index of $1.3$. The analysis of dispersion characteristics requires loss tangent, which is calculated based on the operating frequencies (Veeraselvam et al. 2021a). The loss tangent is varied from $0.0057$ to $0.28$ to check for the absorption characteristics and the same is plotted in Fig. 12a, b for both resonance frequencies. The frequency of the proposed THz sensor appears to have yet to be detuned due to the dispersion properties of the sample. The lossy characteristics of the samples reduce their absorption capabilities. However, at least 66% for law resonance frequency and 43.9% for high resonance frequency absorption is guaranteed for values ranging from $0.0057\mathrm{ to }0.28$.

### Effect of bio-med samples

Coronavirus 2019 (COVID-19) is one of the most serious diseases afflicting many people worldwide. Early diagnosis of this disease is critical in the recovery process. Here, we decided to use the designed THz biosensor to detect coronavirus as well as some other flu viruses, including (H5N1, H5N2, H9N2, H4N6, FAdV) viruses. We show that the proposed THz sensor design can significantly detect these types of viruses with two different EID concentrations and refractive indices (Kuppuswamy et al. 2020). The values of such refractive indices $({n}_{s})$ are $- 0.752147$, $- 0.726881$, $- 0.717555$, $- 0.74535$, $- 0.714207$ and $-0.967251$, corresponding to the H5N1, H5N2, H9N2, H4N6, FAdV and IBV, respectively, and can be rpresented by Bakir et al. (2015):

4 $$\Delta n={n}_{0}-abs({n}_{s})$$

At certain wavlength, the refractive indices of materials are complex in nature (i.e. they have real and imaginary refractive parts). The real refractive indices control the reflected signal while imaginary refractive indices control the material absorbance (Cherkezyan et al. 2012). Refractive indices of different corona viruses (H5N1, H5N2, H9N2, H4N6, FAdV and IBV) have been computed based on reflectance analysis of a virus solution confirming that the negative refractive indices of these viruses 412 nm (Kuppuswamy et al. 2020). Apart from this, the minute observation indicates that the refractive index is around $-0.73$ for all viruses except IBV, which is around $-0.96$. These values are the maximum refractive indices of the coronaviruses. Since the nature of the refractive indices of the IBV, it differs from other viruses, and it belongs to the family of COVID-19, one can realize the status of coronaviruses (whether affected by novel corona or not) by knowing the refractive indices (Kuppuswamy et al. 2020).

**Fig. 13:**
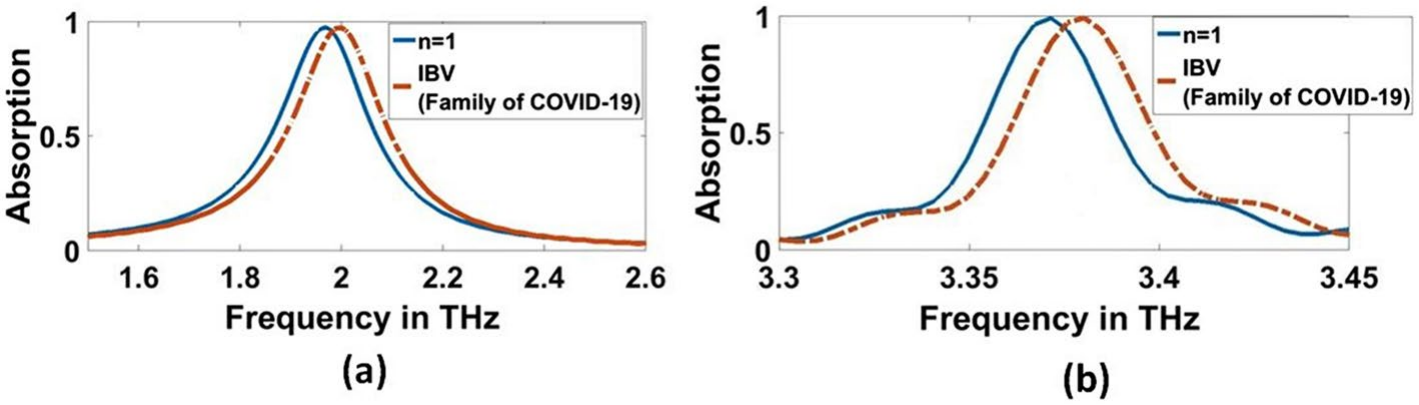
Sensor performance for Coronavirus material characterized by the different indexes of refraction for 0.01 (EID concentration), **a** 1.9656 THz, **b** 3.3692 THz

**Fig. 14:**
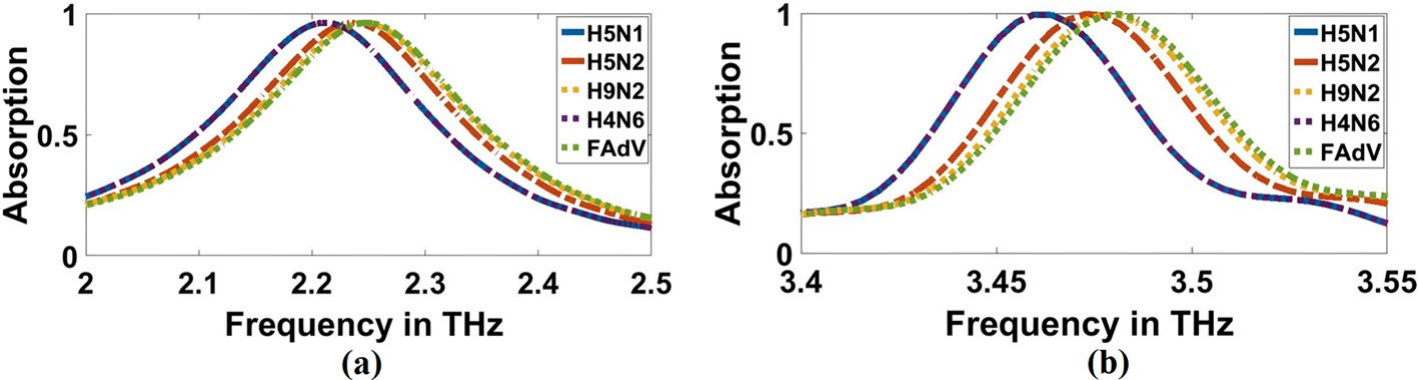
Sensor performance for category of other flus (H5N1, H5N2, H9N2, H4N6, FAdV) characterized by the different indexes of refraction for 0.01 (EID concentration), **a** 1.9656 THz, **b** 3.3692 THz

**Fig. 15:**
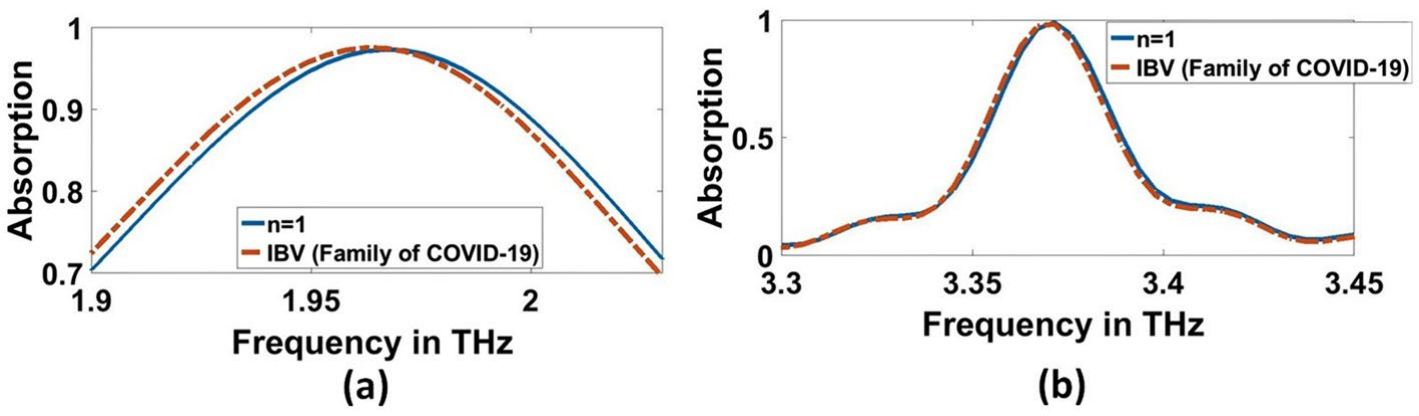
Sensor performance for corona vires material characterized by the different indexes of refraction for 1000 (EID concentration), **a** 1.9656 THz, **b** 3.3692 THz

**Fig. 16:**
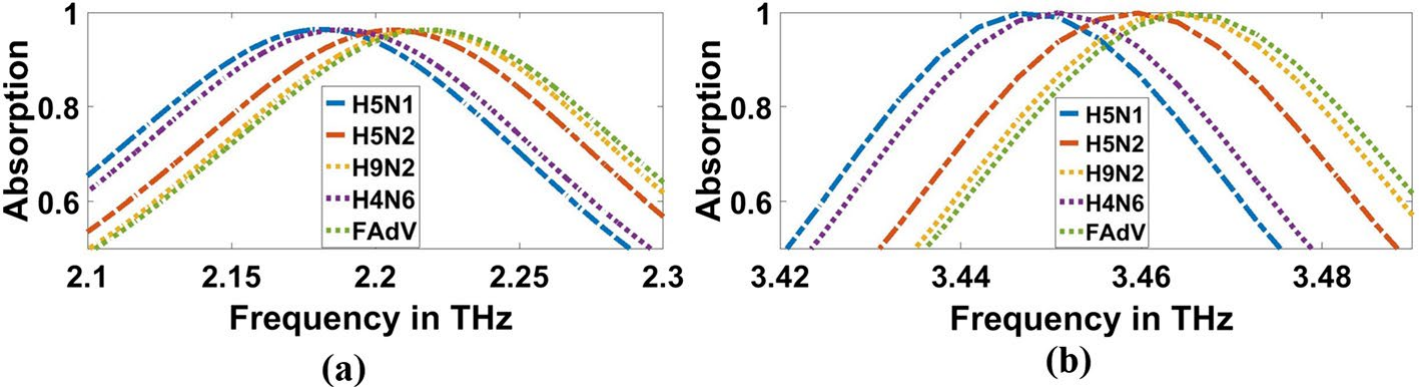
Sensor performance for category of other flus (H5N1, H5N2, H9N2, H4N6, FAdV) characterized by the different indexes of refraction for 1000 (EID concentration), **a** 1.9656 THz, **b** 3.3692 THz

In this step, we overlay a layer with a thickness of $10\mathrm{ \mu m}$ from each virus over the structure and measure the absorption characteristics simulated by the biosensor/detector. According to the results, the absorption characteristics of the biosensor for each of the different viruses at two concentrations of $0.01$ and $1000$ (EID $/5\mathrm{\mu L}$) have a resonance frequency and a different absorption, as shown in Figs. 13, 14, 15 and 16. As presented in Figs. 13a, 14a, and Table 3, the resonant frequency of H5N1, H5N2, H9N2, H4N6, FAdV, and IBV viruses are $2.212$ THz, $2.234$ THz, $2.2428$ THz, $2.212$ THz, $2.2478$ THz, and $1.9964$ THz, respectively, for $0.01$ (EID concentration). Additionally, Figs. 15a, 16a, and Table 4 illustrate the resonant frequency of H5N1, H5N2, H9N2, H4N6, FAd V, and IBV viruses are $2.1812$ THz, $2.2032$ THz, $2.212$ THz, $2.1856$ THz, $2.2164$ THz, and $1.9612$ THz, respectively for $1000$ (EID concentration). According to Figs. 13b, 14b, and Table 3, the resonant frequencies of H5N1, H5N2, H9N2, H4N6, FAdV, and IBV viruses are $3.4611$ THz, $3.4707$ THz, $3.4748$ THz, $3.4572$ THz, $3.4792$ THz, and $3.378$ THz, respectively, for $0.01$ (EID concentration).

**Table 3:** Absorption characteristics for various refractive indexes of Corona Viruses for 0.01 (EID concentration)

Type of viruses	RI	A%	F_r_ THz	Δf (GHz)	FWHM (GHz)	Q-Factor	S, (GHz/RIU)	FoM
Unloaded	1	97.29	1.9656		103.71	19.08	–	–
		99.06	3.3692	–	21.6	155.98		
H5N1	− 0.752147	96.2167	2.212	246.4	111.34	19.867	994.138	8.9288
		99.51	3.4611	91.9	35.291	98.073	370.784	10.506
H5N2	− 0.726881	96.188	2.234	268.4	112.36	19.88	982.7218	8.746
		99.709	3.4707	101.5	35.59	97.519	371.6329	10.44205
H9N2	− 0.717555	96.175	2.2428	277.2	114.47	19.593	981.43	8.574
		99.623	3.4748	105.6	37.131	93.582	373.878	10.069
H4N6	− 0.74535	96.217	2.212	246.4	111.87	19.773	967.60259	8.649
		99.513	3.4572	88	35.295	97.952	345.572	9.791
FAdV	− 0.714207	96.159	2.2472	281.6	113.29	19.836	985.3285	8.697
		99.69	3.4792	110	38.026	91.495	384.894	10.122
IBV (Family of COVID-19)	− 0.967251	97.302	1.9964	30.8	108.86	18.339	940.4867	8.639
		99.136	3.378	8.8	23.953	141.026	268.71	11.218

**Table 4:** Absorption characteristics for various refractive indexes of Corona Viruses for 1000 (EID concentration)

Type of viruses	RI	A%	F_r_ (THz)	Δf (GHz)	FWHM (GHz)	Q-Factor	S, (GHz/RIU)	FoM
Unloaded	1	97.29	1.9656	–	103.71	19.08	–	–
		99.06	3.3692		21.6	155.98		
H5N1	− 0.78266	96.39	2.1812	215.6	108.52	20.099	991.99	9.14
		99.81	3.444	74.8	31.611	108.949	344.16	10.887
H5N2	− 0.75739	96.249	2.2032	237.6	113.74	19.37	979.349	8.61
		99.895	3.4572	88	34.479	100.269	362.722	10.52
H9N2	− 0.74807	96.205	2.212	246.4	112.98	19.579	978.049	8.657
		99.87	3.4616	92.4	34.819	99.417	394.622	11.334
H4N6	− 0.77586	96.327	2.1856	220	113.25	19.633	981.529	8.667
		99.948	3.4484	79.2	32.659	105.588	353.351	10.819
FAdV	− 0.74472	96.283	2.2164	250.8	113.95	19.45	982.45	8.6218
		99.738	3.4616	92.4	34.449	100.485	361.955	10.507
IBV (Family of COVID-19)	− 0.999998	97.499	1.9612	4.4	108.83	18.021	2200 X10^3^	20,215.014
		98.27	3.3692	0	21.754	154.877	0	0

**Fig. 17:**
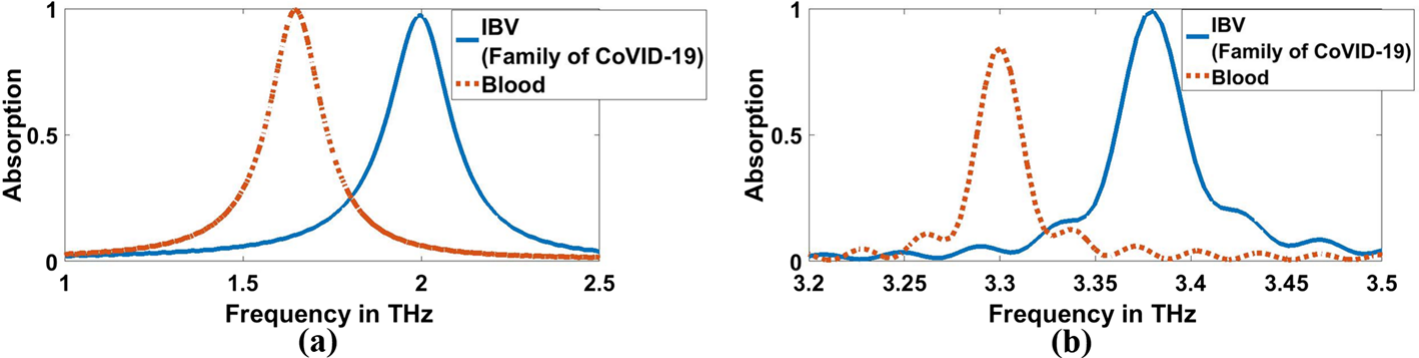
Sensor performance for normal blood sample and coronavirus material characterized by the different indexes of refraction for 0.01 (EID concentration), **a** 1.6444 THz, **b** 3.2988 THz

**Fig. 18:**
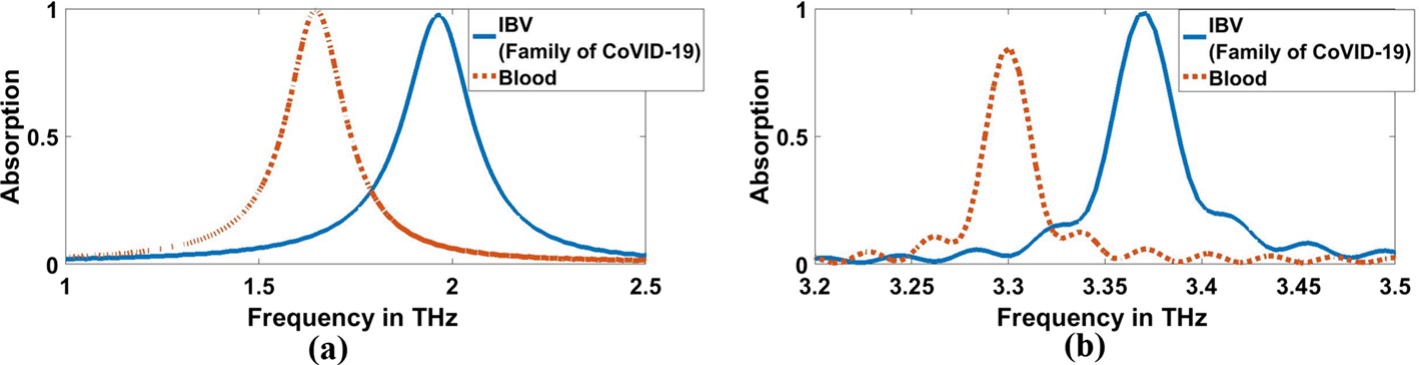
Sensor performance for normal blood sample and corona vires material characterized by the different indexes of refraction for 1000 (EID concentration), **a** 1.6444 THz, **b** 3.2988 THz

Furthermore, Figs. 15b, 16b, and Table 4 clarify the resonant frequencies of H5N1, H5N2, H9N2, H4N6, FAd V, and IBV viruses are $3.444$ THz, $3.4572$ THz, $3.4616$ THz, $3.4484$ THz, $3.4616$ THz, and $3.3692$ THz, respectively for $1000$ (EID concentration), we can detect the corona and category of other flus (H5N1, H5N2, H9N2, H4N6, FAdV) on which it is located. For, H5N1 and H4N6 achieve the same lower resonant frequency, so we can depend on the higher resonant frequency; they achieve $3.4611$ THz and $3.4572$ THz, respectively for $0.01$ (EID concentration), as shown in Fig. 14b and Table 3. The amount of frequency deviation helps to precisely estimate the presence of the type of virus. Furthermore, the absorption in the case of corona virus detection was compared with normal blood. The refractive index of normal blood for is ~ 1.35 (Ma et al. 2020). As illustreated in Figs. 17 and 18, both the lower and higher resonance frequencies of the normal **blood** sample are obviously changed. F_r_ of each frequency is 1.6444 and 3.2988 THz, and Δf = f_corona_–f_blood_ is 0.352, and 0.0792 THz for $0.01$ (EID concentration) as shown in Fig. 17a, b.While in Fig. 18a and b, Δf is 0.3168, and 0.0704 THz for $1000$ EID concentration.

The absorptivity for most of the refractive index shift is seen to be $97\mathrm{\%}$ and $99\mathrm{\%}$ at $1.9656$ THz and $3.3692$ THz, respectively. The theoretical sensitivity of the proposed THz sensor for the refractive index of IBV (Family of COVID-19) is recorded as $2200$ THz/RIU for $1000$ (EID concentration). Table 3 shows the sensor metrics for the proposed THz sensor for $0.01$ (EID concentration). It is clear to observe that the sensor is highly-sensitive for H5N1, with $994.138$ GHz/RIU; however, a relatively lower sensitivity of $940.4867$ GHz/RIU is recorded for IBV (Family of COVID-19) at $1.9656$ THz. While it is highly sensitive for FAdV, with the sensitivity of $384.894$ GHz/RIU, a relatively lesser sensitivity of $268.71$ GHz/RIU is recorded for IBV (Family of COVID-19) at $3.3692$ THz. It is worthy to mention here that the sensitivity was calculated relative to the corona virus and with that n = 1.

The frequency deviation$\mathrm{\Delta f}$, Full-Width Half Maximum (FWHM), Quality factor ($\mathrm{Q}$) and the Figure of Meirt (FoM) are estimated and presented in Tables 3 and 4 for both concentrations. The frequency deviation is greater than $264$ GHz and FWHM is lesser than $114$ GHz at $1.9656$ THz for $0.01$ EID concentrations, while The frequency deviation is greater than $215$ GHz and FWHM is lesser than $113$ GHz at $1.9656$ THz for $1000$ EID concentrations category of other viruses of flu (H5N1, H5N2, H9N2, H4N6, FAdV). The FoM is estimated to be greater than eight at $1.9656$ THz for $0.01$ EID concentration of IBV (Family of COVID-19) with $20215.014$ as a peak value for $1000$ EID concentration of IBV (Family of COVID-19) at the same frequency, but at $3.3692$ THz for $1000$ EID concentration of IBV (Family of COVID-19) there is no frequency shift, so $1.9656$ THz is the better chose for COVID-19.

The width of the star-shaped resonator (a), the length of the star-shaped resonator (b), and the side length (c) of the conductor are the various parameters that are considered to estimate the fabrication tolerance, as shown in Tables 5 and 6 at $1.9656$ THz and $3.3692$ THz, respectively. The results indicate that the proposed biosensor can withstand the fabrication errors by providing absorptivity greater than $94\mathrm{\%}$ and $97\mathrm{\%}$ for $\mathrm{n}=1$ at $1.9656$ THz and $3.3692$ THz, respectively. The estimated sensitivity for $\mathrm{n}=-0.73$ and $\mathrm{n}=-0.96$ is greater than $994$ GHz/RIU and $770$ GHz$/$RIU at $1.9656$ THz for every change in the geometrical parameters of the proposed sensor.

**Table 5:** Investigation of fabrication tolerances for a variety of geometrical parameters at 1.9656 THz

Parameter	Tolerance	Δf )GHz(	% Deviation	Absorption for n = 1	Sensitivity (GHz/RIU) For n = − 0.73	Sensitivity (GHz/RIU) For n = − 0.96
Nano gab A	−5%	0	0	98.406	994.074	880
	+ 5%	4.4	0.2238	94.738	961.4815	770
B	−5%	26.4	1.343	99.99	977.777	990
	+ 5%	48.4	2.46	95.99	994.074	770
C	−5%	22	1.119	99.23	1026.667	880
	+ 5%	26.4	1.343	98.78	1124.445	880

**Table 6:** Investigation of fabrication tolerances for a variety of geometrical parameters at 3.3692 THz

Parameter	Tolerance	Δf (GHz)	% Deviation	Absorption for n = 1	Sensitivity (GHz/RIU) For n = − 0.73	Sensitivity (GHz/RIU) For n = − 0.96
Nano gab a	−5%	4.4	0.1306	98.427	374.8148	220
	+ 5%	4.4	0.1306	99.42	374.8148	220
b	−5%	4.4	0.1306	98.41	374.8148	220
	+ 5%	4.4	0.1306	99.056	374.8148	220
c	−5%	4.4	0.1306	97.87	391.111	220
	+ 5%	22	0.6529	99.36	228.148	110

Table 7 presents a comprehensive performance comparison of other sensors in the literature with the proposed biosensor. The comparison is made using the refractive index for H5N2 and H9N2 viruses of flu viruses. From the Table, it is derived that the proposed sensor achieves higher frequency deviation sensitivity and FoM compared to the reference (Keshavarz and Vafapour 2019).

**Table 7:** Performance comparison with other relevant sensors for H5N2 and H9N2 flus viruses

Refs.	Type of viruses	Performance band THz	F_r_ THz	Δf (GHz)	Sensitivity (GHz/RIU) N_s_ = 1.1	FoM
Keshavarz and Vafapour (2019)	Unloaded H5N2 H9N2	3–0	1.7164 1.665 1.641	51.4 75.4	540	2.86
This work for 0.01 (EID concentration)	Unloaded H5N2 H9N2	3–0	1.9612 2.234 2.2428	268.4 277.2	968	9.46
This work for 1000(EID concentration)	H5N2 H9N2	3–0	2.2032 2.212	237.6 246.4	968	9.46

These results compared with the other THz biosensors in the frequency range of $1-2$ THz (compatible with THz$-$TDS system), and the result of this comparison is proposed in Table 8. It includes ${\mathrm{N}}_{\mathrm{s}}$ and ${\mathrm{H}}_{\mathrm{s}}$ as the refractive index and thickness of analyte, ${\mathrm{F}}_{\mathrm{r}}$ as the resonance frequency of the metamaterial without the analyte, $\mathrm{S}$ for the sensitivity, FWHM, $\mathrm{Q}$ for the quality factor, and FoM. Not only the sensitivity to the sample complex reftractive index (CRI) is increased to ${\mathrm{N}}_{\mathrm{s}}=- 0.999998$, but also there is a remarkable increase in the theoretical sensitivity. So, frequency deviation and resonance frequencies for each virus are important for biosensing, especially in high CRI such as corona and other flu viruses (H5N1, H5N2, H9N2, H4N6, FAdV). Moreover, the FWHM is narrower, leading to a higher Q-factor. Addtionally FOM minimizes the overlapping between the detection thresholds, and makes it an ideal case for biosensing purposes.

**Table 8:** Comparison of sensitivity, Q- factor and FoM in different research works

Refs.	N_s_	H_s_	F_r_ THz	FWHM (GHz)	S, (GHz/RIU)	Q	FoM (RIU)^−1^
Singh et al. (2014)	1.6	16	1.13	–	36	–	–
Rodrlguez-Ulibarri et al. (2016)	2	20	0.7	–	180	0.22	–
Zhang et al. (2018)	1.6	–	0.85	–	182	–	–
Yan et al. (2019)	1.6	11	1.67	400	455.7	–	–
Cheng et al. (2020)	1.8	8	0.81	–	240	3.45	–
Veeraselvam et al. (2021b)	1.33	–	1.85	140	423	13.2	3.02
Nourinovin and Alomainy (2021)	1.6	18	1.94	300	550	6.46	1.8333
This work	1.6	10	1.9656	90.531	836.333	21.711	9.238

Compared with other shaped biosensors, the proposed THz biosensor offers a peak absorption of 97.2% and 99.1% at 1.9656 THz and 3.3692 THz, respectively. The full width at half-maximum corresponding to the absorption frequency is 5.276% and 0.641% repectively and a quality factor for TEM Mode is 19.08 and 155.98 at 1.9656 THz and 3.3692 THz, respectively. Morever, the sensor is angularly independent for angles up to $\uptheta =9{0}^{\mathrm{o}}$ with no degeneration in the absorptivity at $1.9612$ THz which is considered beneficial results over those reported by (Cheng et al. 2018; Veeraselvam et al. 2021a). The peak sensitivity of the proposed sensor is 968 GHz/RIU and 264 GHz/RIU at 1.9656 THz and 3.3692 THz, respectively which is greater than the sensitivity achieved in some relevant literatures (Nourinovin and Alomainy 2021; Yan et al. 2019; Zhang et al. 2018)**.** The proposed biosensor was analyzed and compared with the traditional sensors of a single resonant peak. However, a sensor with two resonant frequencies can significantly reduce the measurement errors and achieve higher accuracy.

Due to polarization independent structure of the presented biosensor, the oblique incidence produces an identical response for TEM, TE, and TM modes of operation. Accordingly, the proposed sensor is insensitive to polarization and angularly independent. Therfore, ensuring same position the structure when testing different concentrations samples in practical applications is not required, so this structure is appropriate for rapid detection. The proposed sensor also provides relatively stable operational characteristics for various sample thicknesses with an average deviation $358.4308$ GHz$/\mathrm{\mu m}$ and $75.8154$ GHz$/\mathrm{\mu m}$ at $1.9656$ THz and $3.3692$ THz, respectively. The sensitivity and frequency deviation show constant linear variation for both resonant. Thus, the frequency deviation study is more appropriate for virus detection.

### Fabrication note

One common technique for the biosensor fabriaction is surface micromachining. In the initial fabrication setp, a negative photoresist layer is used as a coagting for a standard silicon dioxide layer substrate. The composite is then exposed to ultraviolet lamp and mask forming a pattern. Metal evaporation process is then utilized to place a $2\mathrm{ \mu m}$ -thick gold film. The photoresist is finally remoned using the lift-off process which form a metal pattern on the substrate (Li et al. 2021a). Regarding filling the liquids and the liquids set their RI, the liquids trapped by the grooves in upper gold plane, so thickness of the liquids started from a standard silicon dioxide layer. The proposed structures suitable for viruses detection.

## Conclusions

A gold plane perforated star-shaped THz bisensor is proposed for detecting corona and of other flu viruses (H5N1, H5N2, H9N2, H4N6, FAdV). The resonant frequency shift of the biosensor following the deposition of viruses was investigated at two different EID concentrations. The obtained results show that, increasing the refractive index from $1$ to $1.6$ resulted in a decrease in the two resonance frequencies decrease from $1.9612$ to $1.4594$ THz and from $3.3692$ to $3.268$ THz. The resonant frequency shift is higher for the FAdV virus, partly because of its smaller value of the real part of its RI relative to the other subtypes of AI viruses. The senstivity of proposed sensor is $940.4867$ GHz/RIU to IBV (Family of COVID-19) for $0.01$ (EID concentration) and $2200$ THz/RIU to IBV (Family of COVID-19) for $1000$ (EID concentration) at $1.9656$ THz. Additionally, the impact of sample thickness on the sensitivity is analyzed. The maximum obtaiend sensitivity was $968$ GHz/RIU, which is proper value for sensing applications compared to the other related works. Morever, The polarization and angular stability characteristics are evaluated and presented. The results elucidate that the proposed structure is polarization insensitive with angular constancy up to $90$ degrees and no absorptivity degeneration. This would facilate the fabrication process and make the sensor more applicable. For future work, we plan to fabricate the proposed structure with micro and nanofabrication methods and analyze its sensitivity in the characterization of coronaviruses. It will open chances for nano-biosensing metamaterial absorber structures to be utilized for ultrasensitive viruses or microorganism detection in the THz regime.

## Data Availability

All data is presented within this manuscript.
